# xLength: Predicting Expected Ski Jump Length Shortly after Take-Off Using Deep Learning

**DOI:** 10.3390/s22218474

**Published:** 2022-11-03

**Authors:** Johannes Link, Leo Schwinn, Falk Pulsmeyer, Thomas Kautz, Bjoern M. Eskofier

**Affiliations:** Machine Learning and Data Analytics Lab, Department Artificial Intelligence in Biomedical Engineering, Friedrich-Alexander-Universität Erlangen-Nürnberg (FAU), 91052 Erlangen, Germany

**Keywords:** wearable sensors, ultra-wideband, inertial measurement unit, performance prediction, sports analytics, performance analysis

## Abstract

With tracking systems becoming more widespread in sports research and regular training and competitions, more data are available for sports analytics and performance prediction. We analyzed 2523 ski jumps from 205 athletes on five venues. For every jump, the dataset includes the 3D trajectory, 3D velocity, skis’ orientation, and metadata such as wind, starting gate, and ski jumping hill data. Using this dataset, we aimed to predict the expected jump length (xLength) inspired by the expected goals metric in soccer (xG). We evaluate the performance of a fully connected neural network, a convolutional neural network (CNN), a long short-term memory (LSTM), and a ResNet architecture to estimate the xLength. For the prediction of the jump length one second after take-off, we achieve a mean absolute error (MAE) of 5.3 m for the generalization to new athletes and an MAE of 5.9 m for the generalization to new ski jumping hills using ResNet architectures. Additionally, we investigated the influence of the input time after the take-off on the predictions’ accuracy. As expected, the MAE becomes smaller with longer inputs. Due to the real-time transmission of the sensor’s data, xLength can be updated during the flight phase and used in live TV broadcasting. xLength could also be used as an analysis tool for experts to quantify the quality of the take-off and flight phases.

## 1. Introduction

In recent years, the use of tracking systems in sports has become more widespread. As a result, a constantly increasing amount of data are becoming available for analysis. The use of sensors in sports applications was first adopted in a research context, which included studies with small-scale datasets. Over time, wearable devices and sensor systems spread to everyday use and were adapted in professional sports to monitor athletes during training and competition. Thus, data availability increased further, from single runs and actions being analyzed to big data.

Initially, works in the area of sports analytics are mainly based on statistics that are manually extracted during or after the match. Macdonald [1] proposed to train a ridge regression model on variables such as hits, faceoffs, shots, missed shots, blocked shots, and other statistics to predict each player’s contribution to the number of expected goals (xG) in hockey matches. Subsequent work by Rathke [2] translated the approach to soccer and observed that the distance to the goal and the shot angle are the most important variables to predict the xG in soccer. The expected goals metric is a very active field in research [3,4,5,6,7,8] and has found its way into the application. For example, in the Bundesliga, the highest German soccer league, the expected goals are shown in live TV broadcasting for individual goals and scoring moments and their sum as a conclusion of a match [9].

Instead of manually extracting statistics, later work utilized cameras and inertial measurement units (IMUs) to detect and classify activities in sports automatically. Camera-based tracking systems are used for example in ball tracking [10,11,12] and pose estimation [13,14]. However, camera tracking systems are hindered by their expensive cost and are difficult to use under bad weather conditions or in large areas. The other option is sensor-based solutions, which circumvent some of these problems. One main advantage of sensor-based solutions is that they can easily cover large volumes as the sensor is not restricted to a specific area, depending on the technology used. For example, sensors based on IMUs or Global Positioning System (GPS) work independently of reference stations. Sensors based on UWB need a radio connection to reference stations in contrast to a camera-based solution where an unobstructed view between the athlete and the cameras is needed.

Moreover, sensors perform independently from the current weather conditions. Blank et al. [15] used IMUs to detect and classify strokes in table tennis. Kautz et al. [16] explored Deep Neural Networks (DNNs) to classify activities in beach volleyball. Stöve et al. [17] used IMUs to detect individual shots and passes of soccer players with machine learning. Cust et al. [18] provide an overview of model development and performance for machine and deep learning for movement recognition in sports. Best practices have been established in the literature regarding data aggregation, data cleaning, model selection, and other components [16]. Claudino et al. [19] find that out of the 58 studies that they analyzed in their review about injury risk and performance prediction, 26% were about soccer, 22% considered basketball, 10% considered handball, 9% considered Australian football, 9% considered baseball, 9% considered volleyball, 7% considered American football, 5% considered ice hockey, 3% considered rugby, and the remaining 2% addressed field hockey, cricket, and beach volleyball. While a considerable amount of studies have been conducted in the area of performance prediction in sports, some disciplines, such as ski jumping, remain understudied. To the best of our knowledge, no performance prediction study has been conducted in the area of ski jumping so far.

Ski jumping is unique, in contrast to almost all other sports, because there are nearly no amateur athletes. This makes data acquisition generally much more complicated and costly. Therefore, all existing studies on ski jumping tracking are based on relatively small datasets applying various tracking techniques. Elfmark et al. [20] use a differential Global Navigation Satellite System (dGNSS) and video-based pose estimation for performance analysis in ski jumping. They also use the data collected with the dGNSS to determine the aerial phase in ski jumping [21] and assess the steady glide phase [22]. Camera-based tracking was also widely used in ski jumping studies to analyze the take-off [23,24], flight styles [25], ski jumping phases [26,27,28], dynamics [29], and aerodynamic forces [30].

Our research contributes in the following ways. Firstly, we acquired the first large-scale study of ski jumping athletes using wearable sensors, including IMUs and ultra-wideband technology. Secondly and mainly, we contribute the first ski jump length prediction benchmark. Our dataset consists of position, velocity, and skis’ orientation measured during the jumps and additional metadata, including the height of the jumping hill and the weather conditions. We investigate the ability of different deep learning algorithms to predict the jump length of 205 individual athletes on five different ski jumping hills. Specifically, we use a fully connected neural network, two different convolutional neural networks (CNNs), and an LSTM model. Our experiments demonstrate the feasibility of predicting the jumping distances of athletes using deep learning. Here, we investigate the ability of the different models to generalize to unseen athletes and jumping hills. Our results indicate that a pretrained model could be used for new athletes and jumping hills without requiring retraining of the model. Moreover, we explore the behavior of the prediction error with respect to the duration of the time series input and observe a consistent increase in the predictive power of the respective models.

**Figure 1:**
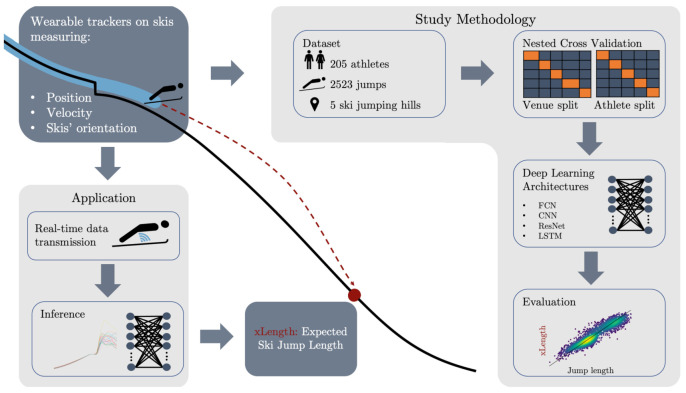
Two wearable trackers are mounted on the athletes’ skis. They measure the 3D position, 3D velocity, and 3D orientations of the skis. In a later application, the data are transmitted in real time and are used to predict the expected jump length (xLength) using deep learning. Additionally, the pipeline of the study methodology is summarized.

A graphical summary of the proposed contribution is shown in Figure 1.

## 2. Materials and Methods

In the following section, the proposed methods are presented. Firstly, the acquired dataset and the respective tracking system are introduced. Consecutively, the deep-learning architectures and their training and hyperparameter tuning are described. Lastly, we introduce the different experiments run in this study.

### 2.1. Dataset

The dataset was acquired using a wearable real-time tracking system (WRTTS). It consists of trackers on top of the athlete’s ski bindings and mobile antennas next to the jumping hill. The WRTTS combines an inertial measurement unit (IMU) with ultra-wideband positioning. Information about the working principle can be found in [31,32,33]. The accuracy of the tracking system has been validated in a previous study [33].

The dataset consists of 2523 jumps acquired during competitions and training of the world’s leading athletes. Every jump includes the 3D trajectory, 3D velocity, and 3D orientation of both skis as well as the wind, wind compensation parameter, gate, gate compensation, and gender. The wind in the dataset is the mean of the tangential wind along the landing hill. This is used in the competitions of the Fédération Internationale de Ski (FIS) to calculate the wind compensation, which is also included in our dataset. The jump length is transformed into points to make competitions fairer, and the wind compensation value is added to compensate for changes in the wind conditions during the competition.

The gate corresponds to the location on the in-run where the athletes start. Since this may change during a competition, this information is crucial since this affects the speed of the athlete at the take-off. Like wind compensation, gate compensation is added to the points of the reached jump length to compensate for changes in the starting gate during a competition.

Additionally, parameters of the ski jumping venues are included and used for the prediction. The venue geometry is described and named according to the ski jumping hill construction standards of the FIS [34]. This involves the height difference (h) between the take-off table edge and the construction point (K), the hill size (HS), the end of landing area (L), the horizontal distance between the edge of the take-off table and K (n), the start of the landing area (P), the height of the take-off table (s), the height difference between the edge of the take-off table and K ($U_{z}$), the inclination of the tangent at P ($\beta_{P}$), the inclination of the tangent at K (β), the inclination of the tangent at L ($\beta_{L}$). Table 1 shows a summary of the acquired dataset.

The jump length within our dataset, which is the regression goal, is determined with the WRTTS and not the manually labeled video distance as in official competitions. Nevertheless, in a previous study, we showed that the jump length determined by the WRTTS and the official video distance differ by 0.31±0.44 m [33].

**Figure 2:**
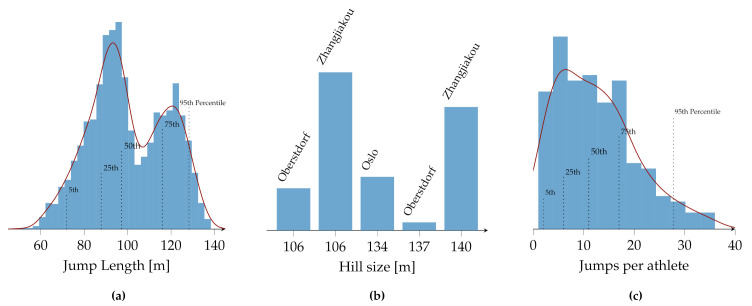
Distribution of the (**a**) jump length, (**b**) jumps per venue, and (**c**) the number of jumps per athlete present in the dataset of this study. The kernel density estimate using Gaussian kernels is also shown in red for the jump length and the jumps per athlete.

Figure 2a shows the distribution of the jump length within the dataset. It ranges from 50.5 to 138.5 m and has two peaks at roughly 93 and 120 m. The median jump length is 97.3 m. In red, the kernel density estimate using Gaussian kernels is depicted.

The data were acquired on five different ski jumping venues ranging from a hill size (HS) of 106 m to 140 m. Figure 2b shows a histogram of the number of jumps per venue with the corresponding HS. The venue with the most jumps is Zhangjiakou, with an HS of 106 m. The ski jumping hill in Zhangjiakou, with an HS of 140 m, has the second most jumps recorded. Both have individually more jumps than the remaining three combined.

Figure 2c shows the distribution of the number of jumps per athlete in the dataset. The median number of jumps per athlete is eleven, and the maximum number is 36 jumps.

**Figure 3:**
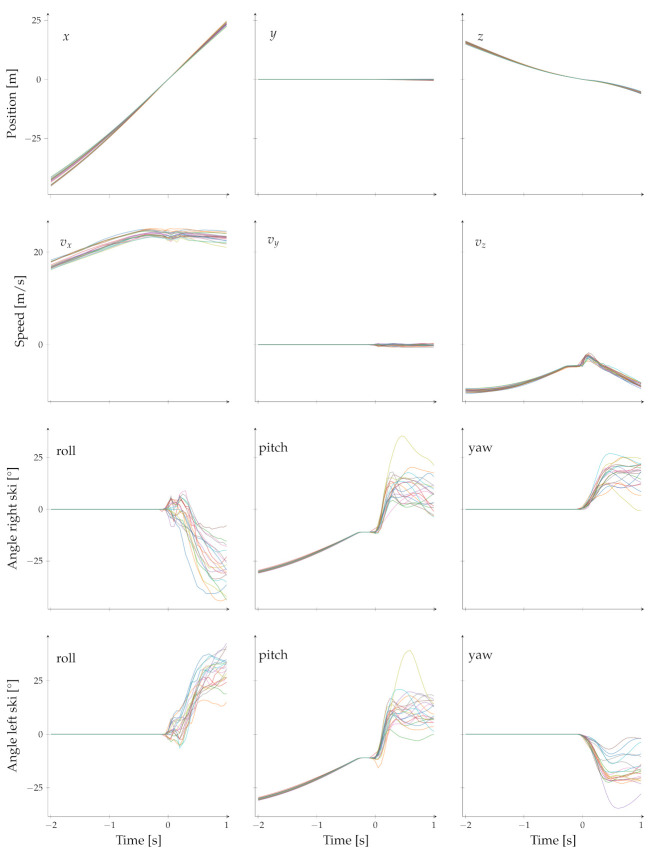
Example data for 20 ski jumps. The figure shows the 3D position, 3D velocity, and the skis’ orientation. The x-axis of the subplots is shared along the columns and the y-axis along the rows.

Figure 3 shows the exemplary position, velocity, and orientation of 20 ski jumps. The data are shown for the time range from two seconds before to one second after the take-off. This time range is also the input for the deep learning architectures to predict the jump length.

The origin of the coordinate system is the edge of the take-off table. With respect to the jumping direction, the x-axis is defined as horizontal, forward, the y-axis as horizontal, left, and the z-axis as vertical, upward.

Before the take-off, the position data are similar for the different jumps due to the tracks along the in-run. The differences only occur due to different venues and varying speeds. Due to the in-run, the $v_{y}$ is 0 before the take-off since the athlete cannot move in the right–left direction.

### 2.2. Deep Learning Architectures

To predict xLength, we tested several deep learning architectures. This includes a fully connected network (FCN), CNN, ResNet [35], and LSTM [36]. With the acquired sensor data, we used the FCN as a baseline to explore the feasibility of predicting the jump length. Furthermore, we explored two CNN-based architectures, a standard CNN and a ResNet, to investigate whether the temporal information in the sensor data improves the generalization of the models to different athletes and venues. Lastly, we used an LSTM to model the temporal aspect of the sensor data more directly and assess if this further improves the regression performance.

As a network input, we used the 3D position, 3D velocity, the orientation of both skis, and the following meta information. As meta information, we used the gender/sex, wind, wind compensation, gate, gate compensation, and the venue-related parameters stated in Table 1. For the FCN, we concatenated all features, including metadata, into a single vector and used it as the input for the neural network. In the case of the CNN and ResNet architecture, we created a two-dimensional vector $x\in R^{N\times C}$, where *N* is the number of sampled sensor values over time, and *C* is the number of different sensor signals (i.e., velocity in the x direction or position in the y direction). Additionally, we concatenated the metadata information to the flattened feature vector after the last convolutional layer of the networks, which is followed by fully connected layers. For the LSTM, we used all the measurements for a single time point as an input and obtained the final prediction by performing a sequential prediction over the whole time series. Here, we also included the metadata information after the last LSTM layer, which is followed by fully connected layers.

The athlete or any past-performance-related features such as ranking in the world cup or previous jumps were not included.

### 2.3. Training and Hyperparameter Tuning

For every deep learning architecture, we performed hyperparameter tuning. We applied a nested 5-fold cross-validation for the hyperparameter tuning using the Bayesian optimization tuning with the Gaussian process implemented in Keras [37]. The nested cross-validation is chosen not to overfit the dataset while performing a hyperparameter tuning and model selection and obtain optimistically biased performance estimations [38]. The search spaces for the different deep learning architectures are depicted in Table 2.

Since the dataset contains strongly dependent data and physical processes, data augmentation techniques are limited and must be chosen carefully. For example, the skis’ orientation affects the aerodynamic drag, which affects the speed and uplift. This again influences the trajectory and the jump length. Therefore, standard augmentation techniques such as random rotation are not applicable.

We, firstly, doubled the number of jumps in the training dataset by mirroring them at the x–z plane. This is equivalent to swapping left and right from the perspective of the ski jumper. Secondly, we added Gaussian noise to all input variables as data augmentation. Apart from these data augmentations, we used standardization as another preprocessing step, i.e., scaling all data to a mean of 0 and a standard deviation of 1.

For the training process, we use the Adam optimizer [39] in combination with a reduction of the learning rate when the loss reaches a plateau. We use the mean squared error as a loss function and terminate the training process via early stopping.

### 2.4. Experiments

Using the previously presented dataset, we perform several experiments. Firstly, we compare the performance of the different deep learning architectures to predict the jump length one second after the take-off. For the comparison, we evaluate the model performances by investigating the following metrics. The residual *r* is, within this work, defined as

(1) $$r=x_{\mathrm{true}}-x_{\mathrm{prediction}},$$

where $x_{\mathrm{prediction}}$ is the ski jump length predicted by a DNN and $x_{\mathrm{true}}$ is the measured ski jump length. To compare the networks’ performance on the whole dataset, we investigate the mean of the residual also called bias

(2) $$\mu =\frac{\sum_{i=1}^{N}x_{\mathrm{true}}^{\left(i\right)}-x_{\mathrm{prediction}}^{\left(i\right)}}{N},$$

where *N* is the number of investigated jumps. Additionally, we analyze the standard deviation of the residual

(3) $$\sigma =\sqrt{\frac{1}{N}\sum_{i=1}^{N}{\left(r^{\left(i\right)}-\mu \right)}^{2}}.$$

The performance of the DNNs is summarized in terms of the mean absolute error (MAE), which is calculated as

(4) $$\mathrm{MAE}=\frac{\sum_{i=1}^{N}\left|x_{\mathrm{true}}^{\left(i\right)}-x_{\mathrm{prediction}}^{\left(i\right)}\right|}{N}.$$

Secondly, we test the generalization capabilities of the different deep learning architectures on unseen athletes and venues. Therefore, we perform the outer split of the nested cross-validation by athletes or venues, respectively. For example, we perform the hyperparameter tuning and model selection on four different ski jumping venues and evaluate this model on the remaining venue.

In addition, we investigate the influence of the input length after take-off on the prediction performance. Since, for longer inputs, the number of weights for the fully connected network would drastically increase; we use a CNN for this experiment. Therefore, we run a hyperparameter tuning for the ResNet for input lengths from 0.5 to 4.0 s after the take-off.

## 3. Results

The following section presents the results obtained from the experiments described in the previous section. We start with comparing the results of the different deep learning architectures for the input interval of two seconds before to one second after the take-off. This also includes investigating the generalization to new athletes and venues. After that, we present the results for the accuracy investigation as a function of the input length.

### 3.1. Prediction of xLength

Table 3 shows the MAE, mean and standard deviation of the prediction error. This includes the folds split by athletes and venues and all tested deep learning architectures. We can see that for the data split by athletes, the ResNet has the best prediction of the jump length with an MAE of 5.3 m, a mean error of 0.1 m and a standard deviation of 6.8 m. The LSTM has the same absolute value for the mean residual with −0.1 m, and the FCN has the same standard deviation of the residual as the ResNet. In general, all architectures achieve similar performance.

Looking at the performances when we split the data by venues, the performances of all architectures decrease in terms of MAE. In addition, the performance of the ResNet decreases in all three metrics. However, the ResNet still has the smallest MAE with 5.9m compared to the other deep learning architectures. The ResNet reaches a mean residual of 0.7m, which is also the smallest value among the different architectures. For the standard deviation of the residual, in contrast, the FCN has the smallest value 7.4m. The ResNet reaches a standard deviation of 7.6m. Especially the LSTM has problems with the generalization over different venues, which results in the largest MAE for the venue split 9.2m.

**Figure 4:**
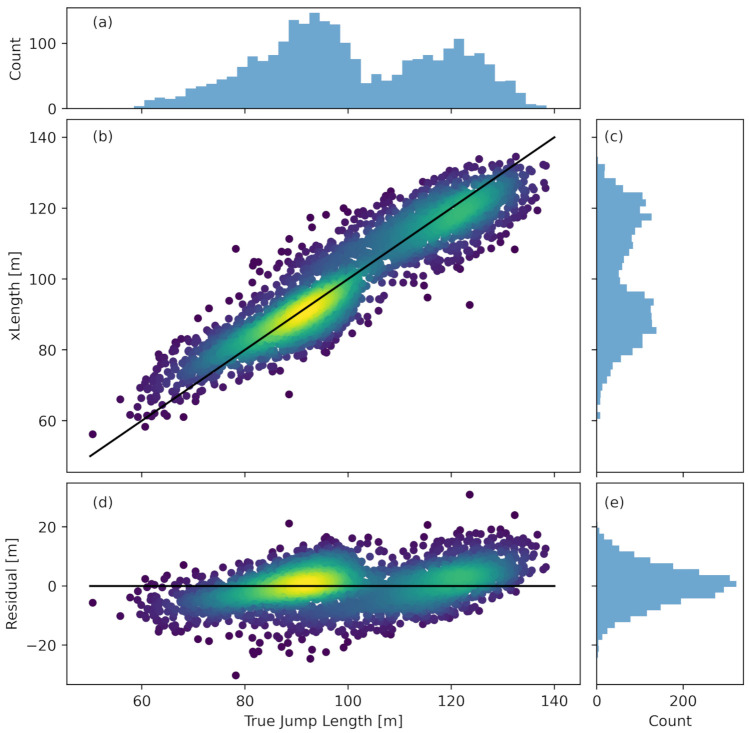
Subplot (**a**) shows the distribution of the true ski jump length in the dataset. Subplot (**c**) shows the distribution of the jump length predicted by the ResNet with the folds split by athletes. In subplot (**b**), the prediction is plotted versus the true jump length. The color represents the density of points calculated using a kernel-density estimate using Gaussian kernels. The brighter the color, the higher the density of the points. Subplot (**d**) shows the residual as a function of the true jump length. The color again represents the density of the points. Subplot (**e**) shows the distribution of the residual.

Figure 4 depicts the predictions of the ResNet with the folds split by athletes. Figure 4b shows the predicted jump length as a function of the true jump length. The color represents the points’ density, and a brighter color corresponds to a higher density. We can see good agreement, so most points lie on or near the line with a perfect prediction. The highest jump lengths are slightly underestimated, and the network overestimates the lowest jump lengths.

Figure 4a shows the distribution of the true jump lengths, and Figure 4c shows the distribution of the predicted jump lengths. Qualitatively, we can see a good agreement between the two distributions.

Figure 4d shows the residual versus the true jump length. The absolute error is relatively constant over the whole span of true jump length, and we do not observe a relative error; i. e., an absolute error increases with increasing jump length. As the left middle plot shows, the predicted jump length at both ends tends toward the mean prediction. The projection of the residual is shown in Figure 4e. The residual follows a Gaussian distribution.

### 3.2. Dependency of Prediction Accuracy on Input Length

In the previous subsection, we investigated the performance of different deep learning architectures in predicting the expected jump length one second after the take-off. In this section, we analyze how the accuracy of the prediction changes with the input length, i.e., the time after the take-off.

**Figure 5:**
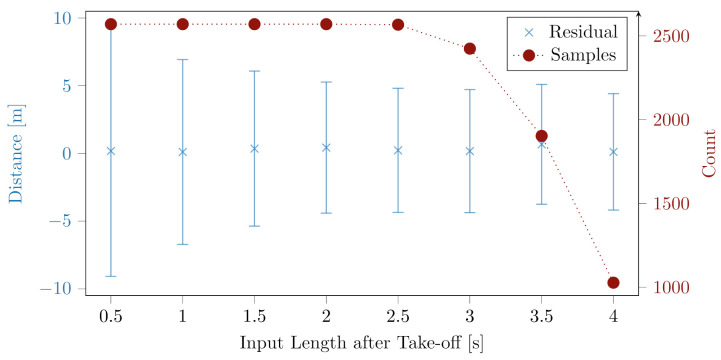
The left y-axis corresponds to the prediction accuracy for ResNets trained on various input lengths after the athlete’s take-off. In blue, the mean of the residual is plotted with the standard deviation of the residual as the y-error bar. Additionally, the number of jumps with a minimum duration, as shown on the x-axis, is plotted in red.

Figure 5 shows the residual for the ResNets trained on different input lengths. The mean residual is depicted as a function of the input length of the time series data. The error bars show the standard deviation of the residual.

The standard deviation of the prediction error decreases from 0.5 to 4.0 s. The mean prediction error is approximately constant for all input lengths.

Additionally, on the right y-axis, the number of jumps in the dataset with a duration longer than the input length of the ResNet is shown. The number of samples is constant until 2.5 s after the take-off. For 3 s after the take-off, the number of samples slightly decreases. For 3.5 s, the number is smaller, and for an input length of 4.0 s after the take-off, there is a factor of more than two fewer jumps than directly after the take-off.

## 4. Discussion

This work aimed to develop the first ski jump length prediction. The dataset and the prediction results are discussed in the following section.

### 4.1. Dataset

The dataset covers a wide range of jump lengths and athletes, which leads to good prediction accuracy over jump lengths and generalization over athletes. Even though the dataset has the largest number of different ski jumping venues and athletes used in a tracking study [21,31,33,40,41,42,43,44], the number of venues is relatively small and unevenly distributed compared to the number of athletes. This could be improved in the future.

Having only sensors on the skis is, on the one hand, unobtrusive and, therefore, perfect for application in competition, especially for world-leading athletes in a dangerous sport such as ski jumping. On the other hand, the tracking system does not cover much information about the athlete’s movement. This would especially be important during the take-off. Therefore, an additional camera next to the take-off table would be beneficial to understand the take-off better and whether an athlete is jumping off too early or too late. For example, pose estimation could be applied to measure knee and hip angles to improve the prediction.

The time series data used in this study were sampled with 20 Hz, which is relatively low considering the high speeds of the athletes at the take-off. The UWB measurements are sampled with 20 Hz, but the internal sampling rate of the IMU sensor is much higher at 1000 Hz. Using this raw and highly sampled IMU data would probably be beneficial for predicting xLength, since the take-off could be analyzed in much more detail. These raw data, unfortunately, were not available in this study.

Future work could consider extending the dataset to ski flying and calculating xLength for ski flying. One challenge is that the data acquisition is even more cumbersome since fewer ski jumping competitions exist.

### 4.2. Prediction of xLength

First of all, we have to emphasize that having a perfect prediction of the ski jump length shortly after the take-off would assume that the remaining flight and landing phase do not influence the jump length, which is, of course, not the case. Therefore, the prediction accuracy of the jump length has a fixed limit. To get as close as possible to this unknown limit is, therefore, the goal of such a prediction. Additionally, since this is the first performance prediction in ski jumping, we cannot compare the prediction results to previous studies.

Checking the generalization to new athletes, all deep learning architectures have a similar prediction performance. The ResNet, however, has the most accurate prediction of xLength in terms of MAE, mean and standard deviation of the prediction error. It could be expected that the generalization to new athletes is no problem for the deep learning architectures since the trajectories differ not much between individual athletes but rather between individual jumps. In addition, the number of athletes is high in the dataset, which benefits this generalization.

All deep learning architectures have worse performance in the MAE for the generalization of new ski jumping venues. It could be expected that the generalization to new venues is worse than to new athletes since the distribution over the venues is not uniform, and the vast majority of jumps are from only two venues. Considering this, the generalization to new venues is better than expected. Additionally, for a possible application, the generalization to new venues should not be a problem since only a few professional ski jumping venues exist, which are thus repeatedly used for competitions. Additionally, acquiring data would be possible during the training runs, which are completed at every venue before the competitions.

Looking at the residual of the ResNet with the data split by athletes (Figure 4), we see that the predictions tend toward the mean jump length. This is not surprising, since the number of samples is much smaller at both ends of the jump length range.

In future work, we will investigate if methods from the area of out-of-distribution generalization can further improve the algorithm’s precision to unseen venues and athletes [45]. Other possibilities include using transfer learning to utilize IMU data from other application areas, where data are more readily available, as completed in earlier work [46].

Another point to mention is that the WRTTS measuring the jump length accuracy is 0.31±0.44 m compared to the official video-based measurement, which is manually labeled and rounded to 0.5 m [33]. Since the deep learning architectures were trained on the jump length, determined with the WRTTS, this would affect a possible application in a competition where the official video-based measurement is used.

In addition, to the prediction of xLength, we also tested interpreting the deep learning models using SHAP (SHapley Additive exPlanations) [47] but did not obtain consistent results over the different folds and deep learning architectures. Future work could further address explainable AI approaches or extracting features to calculate feature importance. This would benefit the athletes, coaches, and sports scientists to better understand the complex sport of ski jumping.

Coaches and sports scientists could use xLength directly after take-off to quantify the quality of the take-off. This would make the evaluation of the take-off objective and, therefore, comparable between athletes.

### 4.3. Dependency of Prediction Accuracy on Input Length

The expected jump length is predicted more precisely with longer inputs, as one would expect, since more flight trajectory data are available and the time to the predicted location is shorter.

Naively, one would expect that the prediction becomes more and more precise. However, the prediction accuracy is almost constant for inputs larger than three seconds. A reason for the performance might be that the jump length is harder to predict due to far fewer data. Additionally, for large jump lengths, which correspond to high jumping durations, the athletes land in the curvature at the bottom of the landing hill, which is not in detail described in the ski jumping hill data input into the network.

Coaches and sports scientists could use xLength of different input lengths to analyze and compare individual jumps. For example, if xLength becomes smaller at a specific time during the jump, this indicates that the athlete made an error during the flight phase. In contrast, if xLength enlarges during the ski jump, this indicates that the athlete performs well in the flight phase. In addition, it might be possible to differentiate between athletes with a better take-off versus athletes with better flight phases.

Additionally, due to the live data transmission of the sensors, the predicted jump length could be updated during the whole jump. In combination with the calculated distance to beat, which is calculated before the athlete starts, this could be used to determine a live probability of reaching a specific position in the ranking or a determination of jump length given away compared to the take-off.

Another possible application could be to use it in a live visualization showing the expected landing corridor, which with increasing prediction accuracy, becomes smaller. So, this feature might, on the one hand, help experts analyze jumps and, on the other hand, make TV broadcasting more interesting for laypersons.

## 5. Conclusions

This work aimed to develop the first ski jump length prediction. Therefore, we analyzed the first large-scale ski jumping dataset included in a research study. The data are measured by wearable trackers on the athletes’ skis, measuring the 3D position, 3D velocity, and the skis’ orientation. Using the ski jump length also determined with the tracking system, we performed a hyperparameter tuning for different deep learning architectures.

Firstly, we compared the performance of the deep learning architectures for a prediction one second after the take-off. Here, we obtain the best results for the generalization to new athletes using a ResNet. This achieves an MAE of 5.3 m, mean residual 0.1 m, and a standard deviation of the residual of 6.8 m. For the generalization to new venues, we obtain slightly different results. Therefore, the ResNet has the smallest MAE with 5.9 m and a standard deviation of the residual of 7.6 m.

Another question was how the prediction accuracy changes with the input time after the take-off. Therefore, we investigated different ResNets trained on various input lengths from 0.5 to 4 s after the take-off. Thereby, the standard deviation of the residual becomes smaller with increasing input lengths.

We think that the proposed xLength can be used for live broadcasting due to the live data transmission and for the retrospective analysis of jumps by experts. This includes the quantification of the take-off, thus comparability between jumps and athletes, as well as the analysis during the flight to determine errors when xLength decreases.

Future work should also consider more explainable approaches than neural networks to obtain a better understanding of ski jumping. Additionally, a camera at the take-off might be beneficial to obtain more information about the take-off process, since the sensors on the skis do not provide any information about the body’s movement.

## Figures and Tables

**Table 1:** Summary of the dataset used in this study. Data were acquired during competition and the training of world-leading athletes. The venue data are named according to the ski jumping hill construction standards of the FIS [34].

Subjects (female/male)	205 (50/155)
Number of jumps	2523
Number of venues	5
Hillsizes (m)	106, 134, 137, 140
Jump length range (m)	50.5 to 138.5
Skill level	Professional
Sampling rate	20 Hz
Time series data	3D position, 3D velocity, 3D orientation of both skis
Athlete data	Athlete ID, gender
Venue data	P, K, HS, L, $U_{z}$, n, h, $\beta_{P}$, β, $\beta_{L}$, s
Jump metadata	Wind, wind compensation, gate, gate compensation

**Table 2:** Intervals of the search spaces during the hyperparameter tuning for the different deep learning architectures. The number of specific layers corresponds to convolutional layers for the CNN, LSTM layers for the LSTM and ResNet blocks.

Hyperparameter	FCN	CNN	ResNet	LSTM
Number of fully connected layers	∈[2,6]	∈[2,6]	∈[2,6]	∈[2,6]
Nodes per fully connected layer	∈[16,272]	∈[16,272]	∈[16,272]	∈[16,272]
Dropout rate	∈[0.0,0.3]	∈[0.0,0.3]	∈[0.0,0.3]	∈[0.0,0.3]
Noise	∈[0.0,0.1]	∈[0.0,0.1]	∈[0.0,0.1]	∈[0.0,0.1]
Batch size	∈[16,272]	∈[16,272]	∈[16,272]	∈[16,272]
Number of specific layers		∈[1,6]	∈[1,6]	∈[1,6]
Filters/LSTM units per layer		∈[16,272]	∈[16,272]	∈[16,272]

**Table 3:** Mean absolute error, mean, and standard deviation of the residual of different deep learning architectures. The prediction of an FCN, CNN, ResNet, and LSTM is tested for nested cross-validation split by athletes and venues.

	Athlete Split			Venue Split		
	**MAE (m)**	**Mean (m)**	**Std (m)**	**MAE (m)**	**Mean (m)**	**Std (m)**
FCN	5.4	0.5	**6.8**	6.0	1.3	**7.4**
CNN	5.5	0.8	6.9	6.1	2.3	7.5
ResNet	**5.3**	**0.1**	**6.8**	**5.9**	**0.7**	7.6
LSTM	5.5	**−0.1**	7.1	9.2	3.8	12.0

## Data Availability

Due to confidentiality agreements, neither the data nor the source of the data can be made available.

## Abbreviations

The following abbreviations are used in this manuscript:

CNN	Convolutional neural network
FCN	Fully connected network
FIS	Fédération Internationale de Ski
h	Height difference between take-off table edge and K
HS	Hill size (distance between edge of take-off table and L)
IMU	Inertial measurement unit
K	Construction point
L	End of landing area
LSTM	Long short-term memory
MAE	Mean absolute error
n	Horizontal distance between take-off table edge and K
P	Start of landing area
s	Height of the take-off table
$U_{z}$	Height difference between take-off table edge and the lowest point
WRTTS	Wearable real-time tracking system
$\beta_{P}$	Inclination of the tangent at P
β	Inclination of the tangent at K
$\beta_{L}$	Inclination of the tangent at L

## References

[1] Macdonald B. An expected goals model for evaluating NHL teams and players. Proceedings of the 2012 MIT Sloan Sports Analytics Conference.

[2] Rathke A. (2017). An examination of expected goals and shot efficiency in soccer. J. Hum. Sport Exerc..

[3] Anzer G., Bauer P. (2021). A goal scoring probability model for shots based on synchronized positional and event data in football (soccer). Front. Sport. Act. Living.

[4] Umami I., Gautama D.H., Hatta H.R. (2021). implementing the Expected Goal (xG) model to predict scores in soccer matches. Int. J. Inform. Inf. Syst..

[5] Bransen L., Davis J. Women’s football analyzed: Interpretable expected goals models for women. Proceedings of the AI for Sports Analytics (AISA) Workshop at IJCAI 2021.

[6] Mackay N. (2017). Predicting Goal Probabilities for Possessions in Football.

[7] Decroos T., Bransen L., Van Haaren J., Davis J. Actions speak louder than goals: Valuing player actions in soccer. Proceedings of the 25th ACM SIGKDD International Conference on Knowledge Discovery & Data Mining.

[8] Robberechts P., Davis J. How data availability affects the ability to learn good xG models. Proceedings of the International Workshop on Machine Learning and Data Mining for Sports Analytics.

[9] Expected Goals (xG) and Goal Probability Explained. https://www.bundesliga.com/en/bundesliga/news/expected-goals-xg-and-goal-probability-explained-13847.

[10] Pingali G., Opalach A., Jean Y. Ball tracking and virtual replays for innovative tennis broadcasts. Proceedings of the 15th International Conference on Pattern Recognition (ICPR-2000).

[11] Gomez G., Herrera López P., Link D., Eskofier B. (2014). Tracking of ball and players in beach volleyball videos. PLoS ONE.

[12] Chen H.T., Tsai W.J., Lee S.Y., Yu J.Y. (2012). Ball tracking and 3D trajectory approximation with applications to tactics analysis from single-camera volleyball sequences. Multimed. Tools Appl..

[13] Badiola-Bengoa A., Mendez-Zorrilla A. (2021). A Systematic Review of the Application of Camera-Based Human Pose Estimation in the Field of Sport and Physical Exercise. Sensors.

[14] Cao Z., Simon T., Wei S., Sheikh Y. Realtime Multi-Person 2D Pose Estimation using Part Affinity Fields. Proceedings of the IEEE Conference on Computer Vision and Pattern Recognition (CVPR).

[15] Blank P., Hoßbach J., Schuldhaus D., Eskofier B.M. Sensor-Based Stroke Detection and Stroke Type Classification in Table Tennis. Proceedings of the 2015 ACM International Symposium on Wearable Computers. Association for Computing Machinery, ISWC ’15.

[16] Kautz T., Groh B.H., Hannink J., Jensen U., Strubberg H., Eskofier B.M. (2017). Activity recognition in beach volleyball using a deep convolutional neural network. Data Min. Knowl. Discov..

[17] Stöve M., Schuldhaus D., Gamp A., Zwick C., Eskofier B. (2021). From the Laboratory to the Field: IMU-Based Shot and Pass Detection in Football Training and Game Scenarios Using Deep Learning. Sensors.

[18] Cust E.E., Sweeting A.J., Ball K., Robertson S. (2019). Machine and deep learning for sport-specific movement recognition: A systematic review of model development and performance. J. Sport. Sci..

[19] Claudino J.G., Capanema D.d.O., De Souza T.V., Serrão J.C., Machado Pereira A.C., Nassis G.P. (2019). Current approaches to the use of artificial intelligence for injury risk assessment and performance prediction in team sports: A systematic review. Sport. Med. Open.

[20] Elfmark O., Ettema G., Groos D., Ihlen E.A., Velta R., Haugen P., Braaten S., Gilgien M. (2021). Performance analysis in ski jumping with a differential global navigation satellite system and video-based pose estimation. Sensors.

[21] Elfmark O., Ettema G., Jølstad P., Gilgien M. (2022). Kinematic determination of the aerial phase in ski jumping. Sensors.

[22] Elfmark O., Ettema G., Gilgien M. (2022). Assessment of the steady glide phase in ski jumping. J. Biomech..

[23] Virmavirta M., Isolehto J., Komi P., Schwameder H., Pigozzi F., Massazza G. (2009). Take-off analysis of the Olympic ski jumping competition (HS-106 m). J. Biomech..

[24] Arndt A., Brüggemann G.P., Virmavirta M., Komi P. (1995). Techniques used by Olympic ski jumpers in the transition from takeoff to early flight. J. Appl. Biomech..

[25] Schmölzer B., Müller W. (2005). Individual flight styles in ski jumping: Results obtained during Olympic Games competitions. J. Biomech..

[26] Chardonnens J., Favre J., Le Callennec B., Cuendet F., Gremion G., Aminian K. (2012). Automatic measurement of key ski jumping phases and temporal events with a wearable system. J. Sport. Sci..

[27] Schwameder H., Müller E., Lindenhofer E., DeMonte G., Potthast W., Brüggemann P., Virmavirta M., Isolehto J., Komi P. (2005). Kinematic characteristics of the early flight phase in ski-jumping. Science and Skiing III.

[28] Virmavirta M., Isolehto J., Komi P., Brüggemann G.P., Müller E., Schwameder H. (2005). Characteristics of the early flight phase in the Olympic ski jumping competition. J. Biomech..

[29] Müller W., Platzer D., Schmölzer B. (1996). Dynamics of human flight on skis: Improvements in safety and fairness in ski jumping. J. Biomech..

[30] Schmölzer B., Müller W. (2002). The importance of being light: Aerodynamic forces and weight in ski jumping. J. Biomech..

[31] Groh B.H., Warschun F., Deininger M., Kautz T., Martindale C., Eskofier B.M. (2017). Automated ski velocity and jump length determination in ski jumping based on unobtrusive and wearable sensors. Proceedings of the ACM on Interactive, Mobile,Wearable
and Ubiquitous Technologies.

[32] Groh B.H., Fritz J., Deininger M., Schwameder H., Eskofier B.M. Unobtrusive and wearable landing momentum estimation in Ski jumping with inertial-magnetic sensors. Proceedings of the 2018 IEEE 15th International Conference on Wearable and Implantable Body Sensor Networks (BSN).

[33] Link J., Guillaume S., Eskofier B.M. (2021). Experimental Validation of Real-Time Ski Jumping Tracking System Based on Wearable Sensors. Sensors.

[34] Gasser H. (2008). Fédération Internationale de Ski (FIS, International Skiing Federation): Skisprungschanzen Baunormen, Oberhofen, CH.

[35] He K., Zhang X., Ren S., Sun J. Deep residual learning for image recognition. Proceedings of the IEEE Conference on Computer Vision and Pattern Recognition.

[36] Hochreiter S., Schmidhuber J. (1997). Long short-term memory. Neural Comput..

[37] Chollet F. (2015). Keras. https://keras.io.

[38] Cawley G.C., Talbot N.L. (2010). On over-fitting in model selection and subsequent selection bias in performance evaluation. J. Mach. Learn. Res..

[39] Kingma D.P., Ba J. (2014). Adam: A method for stochastic optimization. arXiv.

[40] Wilk M., Gebala L., Gepfert M., Drozd M., Kostrzewa M., Piwowar R., Mroszczyk W., Zajac A. (2018). Effect of kinaesthetic differentiation of the in-run position on the jump length in Polish national ski jumpers. Balt. J. Health Phys. Act..

[41] Štepec D., Skočaj D. Video-Based Ski Jump Style Scoring from Pose Trajectory. Proceedings of the IEEE/CVF Winter Conference on Applications of Computer Vision.

[42] Zecha D., Eggert C., Einfalt M., Brehm S., Lienhart R. A convolutional sequence to sequence model for multimodal dynamics prediction in ski jumps. Proceedings of the 1st International Workshop on Multimedia Content Analysis in Sports.

[43] Fang X., Göttlicher C., Holzapfel F. (2018). Attitude estimation of skis in ski jumping using low-cost inertial measurement units. Multidiscip. Digit. Publ. Inst. Proc..

[44] Fang X., Grüter B., Piprek P., Bessone V., Petrat J., Holzapfel F. (2020). Ski jumping trajectory reconstruction using wearable sensors via extended rauch-tung-striebel smoother with state constraints. Sensors.

[45] Schwinn L., Raab R., Nguyen A., Zanca D., Eskofier B.M. Improving Robustness against Real-World and Worst-Case Distribution Shifts through Decision Region Quantification. Proceedings of the Accepted at the International Conference on Machine Learning (ICML), PMLR.

[46] Link J., Perst T., Stoeve M., Eskofier B.M. (2022). Wearable sensors for activity recognition in ultimate frisbee using convolutional neural networks and transfer learning. Sensors.

[47] Lundberg S.M., Lee S.I., Guyon I., Luxburg U.V., Bengio S., Wallach H., Fergus R., Vishwanathan S., Garnett R. (2017). A Unified Approach to Interpreting Model Predictions. Advances in Neural Information Processing Systems 30.

